# OptCom: A Multi-Level Optimization Framework for the Metabolic Modeling and Analysis of Microbial Communities

**DOI:** 10.1371/journal.pcbi.1002363

**Published:** 2012-02-02

**Authors:** Ali R. Zomorrodi, Costas D. Maranas

**Affiliations:** Department of Chemical Engineering, The Pennsylvania State University, University Park, Pennsylvania, United States of America; University of Illinois at Urbana-Champaign, United States of America

## Abstract

Microorganisms rarely live isolated in their natural environments but rather function in consolidated and socializing communities. Despite the growing availability of high-throughput sequencing and metagenomic data, we still know very little about the metabolic contributions of individual microbial players within an ecological niche and the extent and directionality of interactions among them. This calls for development of efficient modeling frameworks to shed light on less understood aspects of metabolism in microbial communities. Here, we introduce OptCom, a comprehensive flux balance analysis framework for microbial communities, which relies on a multi-level and multi-objective optimization formulation to properly describe trade-offs between individual vs. community level fitness criteria. In contrast to earlier approaches that rely on a single objective function, here, we consider species-level fitness criteria for the inner problems while relying on community-level objective maximization for the outer problem. OptCom is general enough to capture any type of interactions (positive, negative or combinations thereof) and is capable of accommodating any number of microbial species (or guilds) involved. We applied OptCom to quantify the syntrophic association in a well-characterized two-species microbial system, assess the level of sub-optimal growth in phototrophic microbial mats, and elucidate the extent and direction of inter-species metabolite and electron transfer in a model microbial community. We also used OptCom to examine addition of a new member to an existing community. Our study demonstrates the importance of trade-offs between species- and community-level fitness driving forces and lays the foundation for metabolic-driven analysis of various types of interactions in multi-species microbial systems using genome-scale metabolic models.

## Author Summary

Microorganisms rarely live isolated in their natural environments but rather function in consolidated and socializing communities. Despite the growing availability of experimental data, we still know very little about the metabolic contributions of individual species within an ecological niche and the extent and directionality of interactions among them. This calls for development of efficient modeling frameworks to shed light on less understood aspects of metabolism in microbial communities. Here, we introduce OptCom, a comprehensive mathematical framework for metabolic modeling and analysis of microbial communities, which relies on a multi-level/objective optimization formulation to properly describe trade-offs between individual vs. community level fitness criteria. OptCom is general enough to capture any type of interactions (positive, negative or combinations thereof) and is capable of accommodating any number of microbial species involved. We first demonstrate the capability of OptCom to quantify known metabolic interactions in a well-characterized microbial community. We next apply it to more complex communities to assess the optimality levels of growth for each microorganism, elucidate the extent and direction of inter-species metabolite transfers and examine addition of a new member to an existing community. Our study lays the foundation for metabolic-driven analysis of various types of interactions in multi-species microbial systems.

## Introduction

Solitary species are rarely found in natural environments as most microorganisms tend to function in concert in integrative and interactive units, (i.e., communities). Natural microbial ecosystems drive global biogeochemical cycling of energy and carbon [1] and are involved in applications ranging from production of biofuels [2], [3], biodegradation and natural attenuation of pollutants [4], [5], [6], bacterially mediated wastewater treatment [7], [8] and many other biotechnology-related processes [9], [10]. The species within these ecosystems communicate through unidirectional or bidirectional exchange of biochemical cues. The interactions among the participants in a microbial community can be such that one or more population(s) benefit from the association (e.g., through cooperation), some are negatively affected, (e.g., by competing for limiting resources), or more often than not a combination of both. These inter-species interactions and their temporal changes in response to environmental stimuli are known to significantly affect the structure and function of microbial communities and play a pivotal role in species evolution [11], [12], [13], [14], [15], [16].

Recent advances in the use of high-throughput sequencing and whole-community analysis techniques such as meta-genomics and meta-transcriptomics promise to revolutionize the availability of genomic information [16], [17], [18]. Despite the growing availability of this high-throughput data, we still know very little about the metabolic contributions of individual microbial players within an ecological niche and the extent and directionality of metabolic interactions among them. This calls for development of efficient modeling frameworks to elucidate less understood aspects of metabolism in microbial communities. Spurred by recent advances in reconstruction and analysis of metabolic networks of individual microorganisms, a number of metabolic models of simple microbial consortia have been developed. Efforts in this direction started with the development of metabolic model for a mutualistic two-species microbial community [19]. The metabolic network of each microorganism was treated as a separate compartment in analogy to eukaryotic metabolic models [20], [21]. A third compartment was also added through which the two organisms can interact by exchanging metabolites. The same approach was employed for the metabolic modeling of another syntrophic association between *Clostridium butyricum* and *Methanosarcina mazei* [22]. Lewis *et al* [23] have also described a workflow for large-scale metabolic modeling of interactions between various cell lines in the human brain using compartments to represent different cells. Similarly, Bordbar *et al* [24] developed a multi-tissue type metabolic model for analysis of whole-body systems physiology. Alternatively, others proceeded to identify and model synthetic interactions among different mutants of the same species using genome-scale metabolic models. For example, Tzamali *et al* [25] computationally identified potential communities of non-lethal *E. coli* mutants using a graph-theoretic approach and analyzed them by extending dynamic flux balance analysis model of Varma and Palsson [26]. The same researchers have recently extended their study to describe the co-growth of different *E. coli* mutants on various carbon sources in a batch culture [27]. Wintermute and Silver [28] identified mutualistic relationships in pairs of auxotroph *E. coli* mutants. Each pair was modeled using an extended form of the minimization of metabolic adjustment (MOMA) hypothesis [29]. More recently, the concept of inducing synthetic microbial ecosystems not by genetic modifications but rather with environmental perturbations such as changing the growth medium was introduced [30].

All these studies aimed primarily at modeling communities where one or both species benefit from the association while none is negatively affected. The first study to characterize a negative interaction between two microorganisms using genome-scale metabolic models was published by Zhuang *et al* [31] where similar to [25], [27] an extension of the dynamic flux balance analysis [32] was employed to model the competition between *Rhodoferax ferrireducens* and *Geobacter sulfurreducens* in an anoxic subsurface environment. The same procedure was also employed in a study that characterized the metabolic interactions in a co-culture of *Clostridium acetobutylicum* and *Clostridium cellulolyticum* [33]. A wide range of methods beyond flux balance analysis have been used to model microbial communities [34], [35], [36], [37], [38], [39], [40], [41], [42], [43], [44], [45]. For example, Taffs *et al* [46] proposed three different approaches based on elementary mode analysis to model a microbial community containing three interacting guilds. Other studies have drawn from evolutionary game theory, nonlinear dynamics and the theory of stochastic processes to model ecological systems [39], [40], [43].

Despite these efforts, all existing methods for the flux balance analysis of microbial communities are based on optimization problems with a single objective function (related to individual species), which cannot always capture the multi-level nature of decision-making in microbial communities. For example, the flux balance analysis model described in [19] is applicable only to syntrophic associations, where the growth of both species is coupled through the transfer of a key metabolite. The dynamic flux balance analysis models introduced by Zhuang *et al* [31] and Tzamali *et al* [25], [27] rely on solving separate FBA problems for each individual species within each time interval. In all cases these methods cannot trade off the optimization of fitness of individual species versus the fitness function of the entire community. Therefore, there is still a need to develop an efficient modeling procedure to address this issue and to analyze and characterize microbial communities of increasing size with any combination of positive and/or negative interactions.

Here, we introduce OptCom, a comprehensive flux balance analysis framework for microbial communities, which relies on a multi-level optimization description. In contrast to earlier approaches that rely on a single objective function, OptCom's multi-level/objective structure enables properly assessing trade-offs between individual vs. community level fitness criteria. This modeling framework is general enough to capture any type of interactions (positive, negative or combination of both) for any number of species (or guilds) involved. In addition, OptCom is able to explain *in vivo* observations in terms of the levels of optimality of growth for each participant of the community. We first analyze a simple and well-determined microbial community involving a syntrophic association between *D. vulgaris* and *M. maripaludis* [19] to demonstrate the ability of OptCom in recapitulating known interactions. Next, OptCom is employed to model the more complex ecological system of the phototrophic microbial mats of Octopus and Mushroom Springs of Yellowstone National Park and compare our results with those obtained using elementary mode analysis [46]. OptCom identifies the level of sub-optimal growth of one of the guilds (SYN) in this community to benefit other community members and/or the entire population. Finally, we use OptCom to elucidate the extent and direction of inter-species metabolite transfers for a model microbial community [47], identifying the proportion of metabolic resources apportioned to different community members and predicting the relative contribution of hydrogen and ethanol as electron donors in the community. Addition of a new member to this community is also examined in this study.

## Methods

**Figure 1:**
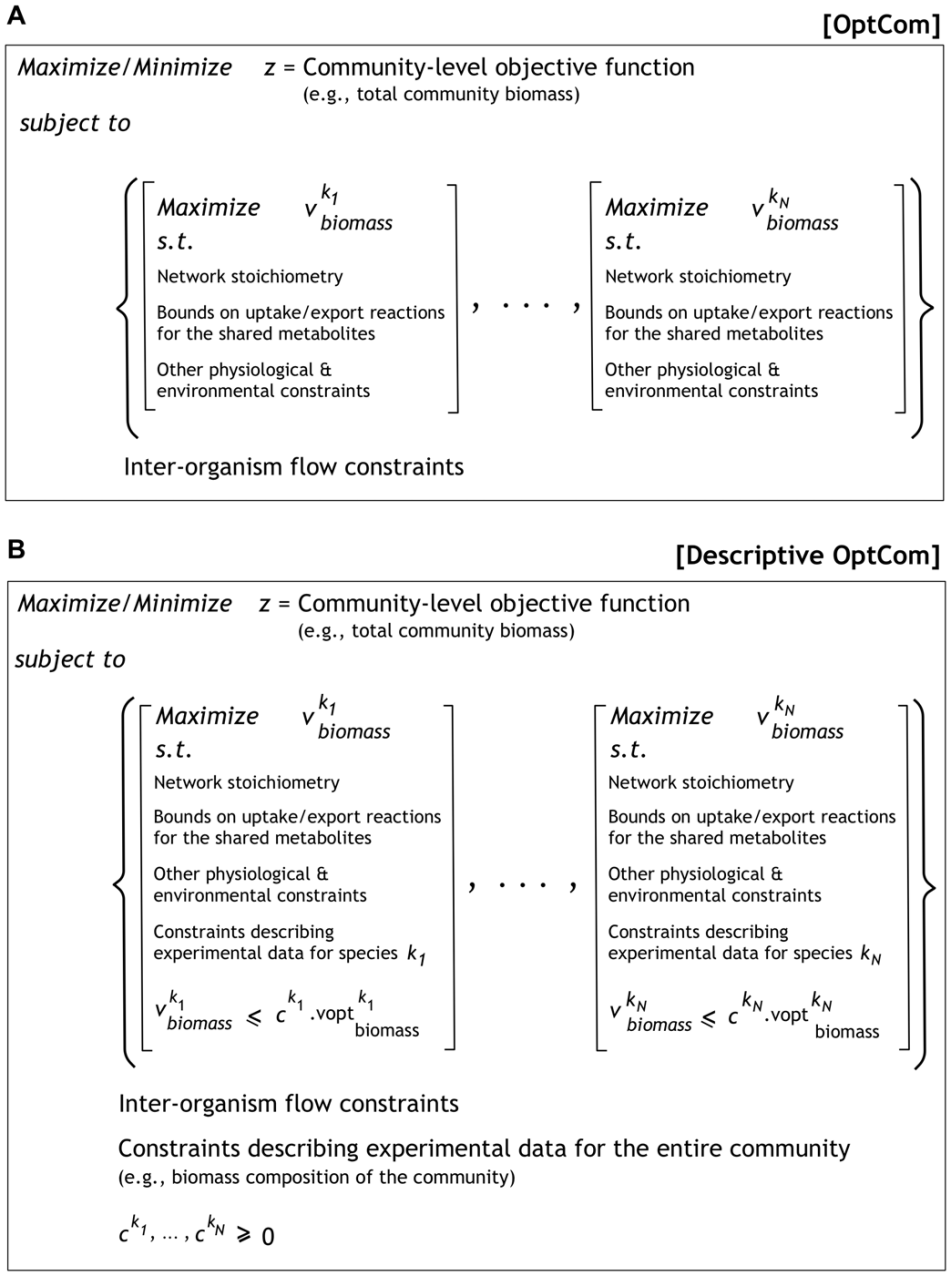
Schematic illustration of OptCom. (A) The multi-level optimization structure of the OptCom. A separate biomass maximization problem is defined for each species as inner problems. These inner problems are then integrated in the outer stage through the inter-organism flow constraint to optimize a community-level objective function. (B) Structure of the Descriptive OptCom to determine the optimality level of each species (*c^k^*), given a set of experimental data. The available experimental data for the entire community and the individual species are described using constraints in the outer and inner problems, respectively, whereas, sub- or super-optimal behavior of each microorganism is captured by using a constraint for the respective inner problem.

OptCom postulates a separate biomass maximization problem for each species as inner problems. The inner problems capture species-level fitness driving forces exemplified through the maximization of individual species' biomass production. If preferable, alternate objective function (e.g., MOMA [29]) could be utilized in the inner stage to represent the cellular fitness criteria. Inter-species interactions are modeled with appropriate constraints in the outer problem representing the exchange of metabolites among different species. The inner problems are subsequently linked with the outer stage through inter-organism flow constraints and optimality criteria so as a community-level (e.g., overall community biomass) objective function is optimized. Figure 1A schematically illustrates the proposed concept. OptCom is solved using the solution methods previously developed for bilevel programs [48], [49], [50], [51] (see Text S1 for details of the optimization formulation and solution). Note that since OptCom yields a (non-covex) bilinear optimization problem, all case studies presented in this paper were solved using the BARON solver [52], accessed through GAMS, to global optimality.

It is important to note that OptCom can be readily modified to account for the case when one or more organisms show a form of cooperative behavior that benefits the whole population, but comes at the expense of growing at rates slower than the maximum possible [15], [53]. To quantify the deviation of community members from their optimal behavior, we introduce a metric called *optimality level* for each species *k* (i.e., *c^k^*). The optimality level for each one of the microorganisms is quantified using a variation of OptCom which we refer to as *descriptive*. Descriptive OptCom incorporates all available experimental data for the entire community (e.g., community biomass composition) as constraints in the outer problem and all data related to individual species as constraints in the respective inner problems while allowing the biomass flux of individual species to fall below (or rise above) the maxima (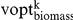) of the inner problems (see Figure 1B). We note that here the optimum biomass flux for each species (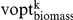) is community-specific as it is computed in the context of all microorganisms striving to grow at their maximum rate (using the formulation given in Figure 1A). An optimality level of less than one for a microorganism *k* implies that it grows sub-optimally at a rate equal to 100*c^k^* % of the maximum (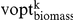) to optimize a community-level fitness criterion while matching experimental observations. Alternatively, an optimality level of one implies that microorganism *k* grows exactly optimally at a rate equal to 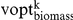 whereas a value greater than one indicates that it achieves a higher biomass production level than the community-specific maximum (i.e., super-optimality) by depleting resources from one or more other community members. It is worth noting that super-optimality is achievable for a species only at the expense of sub-optimal behavior of at least one other member in the community. The identified combination of sub- and/or super-optimal behaviors of individual species is driven by the maximization of a community-level criterion (e.g., maximize the total community biomass).

**Figure 2:**
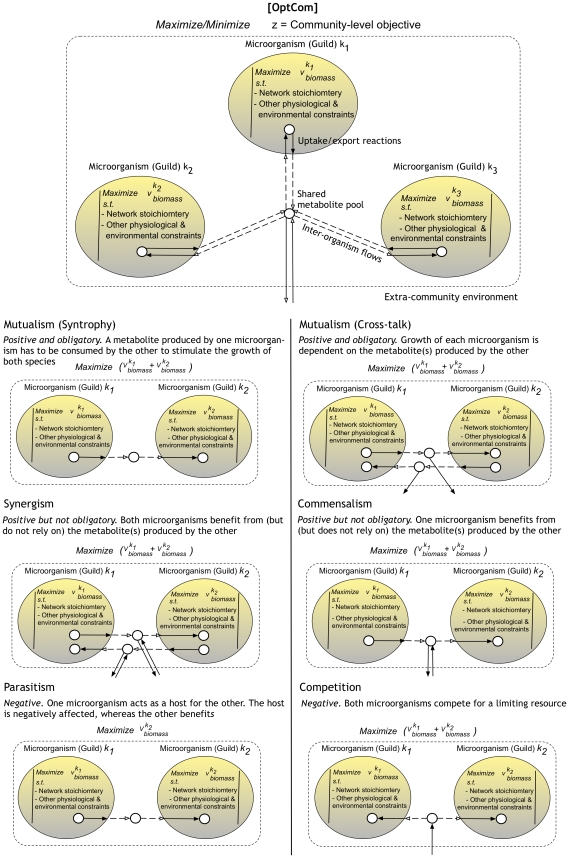
Pictorial illustration of the customized OptCom for various types of interactions. OptCom (top panel) can be readily customized for each type of interaction through properly adjusting the inter-organism flow constraints as demonstrated for a typical microbial community composed of two interacting members.

OptCom can capture various types of interactions among members of a microbial community. Symbiotic interactions between two (or more) populations can be such that one or more species benefit from the association (i.e., *positive* interaction), are negatively affected (i.e., *negative* interactions), or combination of both. Mutualism, synergism and commensalism are examples of positive interactions, whereas parasitism and competition are examples of negative interactions. A pictorial representation of how these interactions can be captured within OptCom by appropriately restricting inter-organism metabolic flows is provided in Figure 2 (see Text S1 for implementation details).

## Results

### Modeling a mutualistic microbial community

We first explore the capability of OptCom to model and analyze a relatively simple and well-characterized syntrophic association between two microorganisms, namely *Desulfovibrio vulgaris* Hildenborough and *Methanococcus maripaludis*. Syntrophy is a mutualistic relationship between two microorganisms, which together degrade an otherwise indigestible organic substrate. A prominent example of syntrophic interactions is interspecies hydrogen transfer, where the hydrogen produced by one of the species has to be consumed by the other to stimulate the growth of both microorganisms [54], [55], [56], [57]. In these communities degradation of a substrate by fermenting bacteria is energetically unfavorable as it carries out a reaction, which is endergonic under standard conditions. However, if this fermenting bacteria is coupled with a hydrogen scavenging partner such as methanogenic bacteria, the organic compound degrading reaction can proceed [58]. Methanogens use hydrogen and energy gained from the first reaction and reduce CO_2_ to methane [56], [58].

Here we focus on such a syntrophic association between *Desulfovibrio vulgaris* Hildenborough and *Methano- coccus maripaludis* S2, for which genomes-scale metabolic models as well as experimental growth data for the co-culture are available [19]. With lactate as the sole carbon source and in the absence of a suitable electron acceptor for the sulfate reducer, *M. maripaludis* provides favorable thermodynamic conditions for the growth of *D. vulgaris* by consuming hydrogen and maintaining its partial pressure low. Stoylar *et al* [19] modeled this microbial community as a multi-compartment metabolic network and employed FBA to identify community-level fluxes by maximizing the weighted sum of the biomass fluxes of two microorganisms.

#### Comparing the OptCom predictions with experimental results

First, we examined whether our model is capable of predicting the relative abundance of species (i.e., composition) in the community by maximizing the community biomass as the outer problem objective function. Each microorganism was allowed to maximize its own biomass yield in the inner problems. Consistent with Stoylar *et al* [19], the lactate uptake rate was set to 48 µM/h and formate and hydrogen accumulation were set to zero, so as all formate and hydrogen produced by *D. vulgaris* is utilized by *M. maripaludis*. Lower and upper bounds on all other reactions (except for the uptake and export fluxes of the shared metabolites) were taken from [19]. The ratio of the biomass yields for *D. vulgaris* and *M. maripaludis* was predicted to be 2.28 based on our simulations. This is consistent with *in vivo* observation that *D. vulgaris* dominates in the co-culture by a ratio of at least 2∶1 [19]. Throughout this and the following studies we assume that the biomass flux for each species is proportional to its biomass abundance in the community.

**Figure 3:**
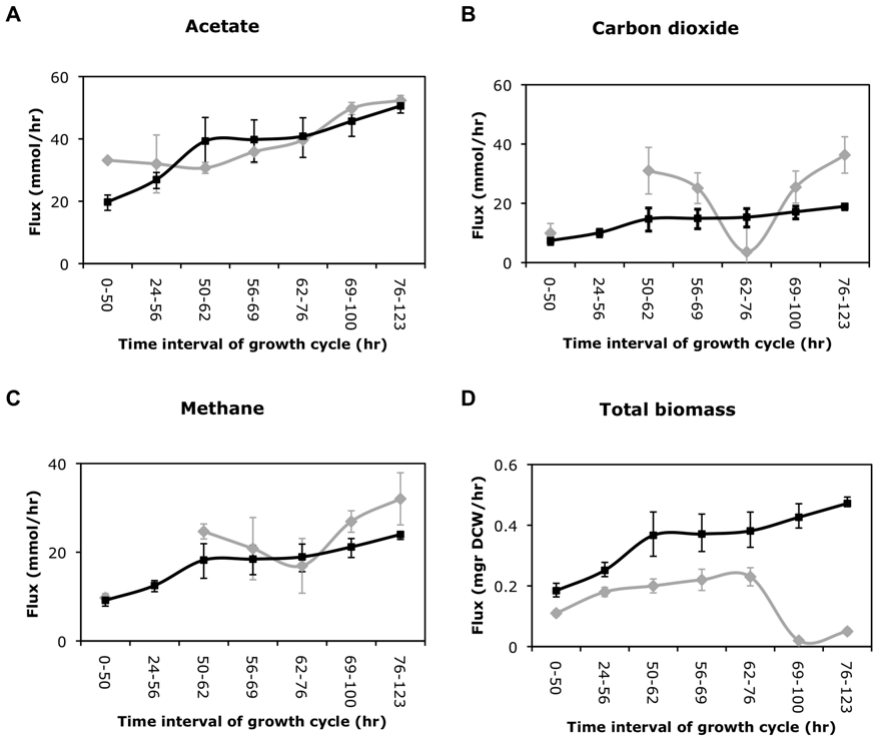
Comparison of the predicted metabolic activities during the syntrophic growth with experimental data. Experimentally determined (gray diamond) and predicted production fluxes by OptCom (black square) for (A) acetate, (B) carbon dioxide (C) methane and (D) total community biomass in the syntrophic growth of *D. vulgaris* and *M. maripaludis*. All experimental data were obtained through personal communications with authors of [19]. A separate simulation was performed for each time interval wherein lactate uptake and hydrogen evolution rates were fixed at their experimentally determined values for that interval. Error bars for experimental values indicate the bounds of 95% confidence intervals [19]. The error bars for OptCom predictions were calculated by performing the simulations on the upper and lower bounds of the 95% confidence intervals for measured lactate and hydrogen flux rates.

We next explore how well OptCom performs in predicting various metabolic activities across different stages of syntrophic growth. To this end, we applied OptCom for each time interval and compared the model predictions for acetate, methane and carbon dioxide evolution rates as well as total biomass production rates with experimental measurements [19]. A separate run was performed for each time interval where lactate uptake and hydrogen evolution rates were fixed at their experimentally determined values in that interval [19]. The results of this comparison are illustrated in Figure 3. We can see that OptCom predictions are generally in good agreement with experimental data especially for the acetate and methane production rates. The predicted CO_2_ evolution rate, however, is lower in all time intervals (except for 62–76 hr) than the measured values. Between 62 hr and 76 hr the experimental data show that the CO_2_ evolution rate is close to zero, which may indicate that all CO_2_ produced by *D. vulgaris* is consumed by *M. maripaludis* [19]. In addition, OptCom predicts an increase in the biomass production of the whole community over time with increasing lactate uptake rate as expected, although, all of predicted yields are higher than experimental measurements. This inconsistency could be due to missing regulatory information, incorrect modeling of ATP utilization and maintenance energy requirements and/or the presence of futile cycles in the metabolic models of one or both species. It is worth noting that all predictions by Stolyar's multi-compartment approach are also very close to the results obtained by OptCom. This is because in this syntrophic microbial community the growth of both species is coupled and uniquely dependent on the exchange of hydrogen and/or formate. This allows for a single fitness function to describe the behavior of the entire community.

#### The role of hydrogen and formate in interspecies electron transfer

Hydrogen and formate are primary shuttle compounds for interspecies electron transfer. There are two enzymes in D. vulgaris that are involved in production of hydrogen and formate namely pyruvate oxidoreductase and pyruvate-fomrate lyase [19], [59]. While both of these enzymes convert pyruvate to acetyl-CoA, the former produces reduced ferredoxin, which is then used for hydrogen production, whereas the latter produces formate, which can be secreted to the medium. For an uptake rate of 10 µmol/hr, OptCom predicts that a total of 18.6 µmol/hr of electron transfer in the form of hydrogen and/or formate transfer are required to achieve the maximum growth for both species and community. To investigate the relative contribution of formate and hydrogen in interspecies electron transfer, we examined what portion of the total required electron transfer could be carried by hydrogen or formate while maintaining the maximum biomass yield for both species. This analysis showed that hydrogen could be used as the sole electron carrier to support the maximum growth for both microorganisms even if no formate is secreted by D. vulgaris. Formate, on the other hand, could only account for up to 26% (4.9 µmol/hr) of the total electron transfer to maintain the biomass productions at their maximum. In addition, OptCom results show that formate exchange rates of more than 5.5 µmol/hr (∼30%) are not able to support growth for any of the two species. Using OptCom we find that D. vulgaris is unable to produce sufficient formate to meet the minimum electron transfer required to maintain the redox balance in the absence of hydrogen.

When hydrogen production by *D. vulgaris* is constrained to be at most 13.7 µmol/hr (i.e., the rest of 4.9 µmol/hr electron transfer is assumed to be carried out by formate if possible), OptCom predictions show that in a co-culture consisting of *D. vulgaris* and a mutant of *M. maripaludis* the growth rate of both *D. vulgaris* and *M. maripaludis* is reduced by 26%. The simulation results also show that no fomrate is produced by *D. vulgaris* in this case, which was expected, as it cannot be consumed by the *M. maripaludis* mutant. Despite no formate production by *D. vulgaris*, OptCom reveals that the flux through pyruvate formate lyase is higher compared to the community having the wild-type strains. Further investigation of the *in silico* flux distributions shows that the entire amount of formate produced by the pyruvate formate lyase reaction is directed towards CO_2_ production. This in turn results in an increased consumption of CO_2_ by the *M. maripaludis* mutant and consequently a lower accumulation of CO_2_ in the extracellular environment compared to the community with the wild-type strains. The predictions by OptCom for the community with mutant of *M. maripaludis* are in agreement with experimental results by Stolyar *et al* [19] who established a syntrophic association between *D. vulgaris* and the *M. maripaludis* mutant MM709 lacking the two annotated formate dehydrogenase enzymes. It was observed that this co-culture is able to grow, confirming that hydrogen alone can support the syntrophic growth of both species. Nevertheless, the growth rate, biomass yield and lactate uptake rates were lower compared to the syntrophic growth between the wild-type strains [19]. Notably, OptCom predictions suggest that if the wild-type *D. vulgaris* in Stolyar's experiment is replaced with a mutant lacking pyruvate-formate lyase, so as all electron equivalent is produced in the form of hydrogen, then the co-culture should be able to restore growth to that of wild-type species community as hydrogen alone can carry all required electron equivalents.

### Assessing optimality levels in a phototrophic microbial community

Here we examine the applicability of OptCom for modeling a more complex microbial community containing three interacting guilds, the phototrophic microbial mats of Octopus and Mushroom Springs of Yellowstone National Park (Wyoming, USA) [60]. The inhabitants of this community include unicellular cyanobacteria related to *Synechococcus* spp (SYN), filamentous anoxygenic phototrophs (FAP) related to *Chloroflexus* and *Roseiflexus* spp and sulfate-reducing bacteria (SRB) as well as other prokaryotes supported by the products of the photosynthetic bacteria [46], [60]. Diel (day-night) variations in metabolic activities of members of this community were observed before [61], [62], [63]. During the day when the mat is oxygenated cyanobacteria appear to be the main carbon fixer, consuming CO_2_ and producing storage products such as polyglucose as well as O_2_ as a by-product of photosynthesis. High levels of O_2_ relative to CO_2_ stimulate the production of glycolate. Glycolate is then used as a carbon and energy source by other community members such as photoheterotrophic FAP. At night, the mat becomes anoxic and cyanobacteria start to ferment the stored polyglucose into small organic acids such as acetate, propionate and H_2_. FAP can incorporate fermentation products photoheterotrophically while SRB oxidizes the fermentation products under anaerobic condition and produces sulfide [60], [64], [65], [66]. A schematic diagram representing the interactions in this community is given in [46].

This microbial community has been previously modeled and analyzed by Taffs *et al* [46] using a representative microorganism for each guild: Oxygenic photoautotrophs related to *Synechococcus* spp were chosen to represent the mat's primary carbon and nitrogen fixers. FAP from the family *Chloroflexaceae*, were selected to represent metabolically versatile photoheterotrophs that capture light energy as phosphodiester bonds but require external reducing equivalents and carbon sources other than CO_2_. A SRB guild representative whose metabolic behavior was based on several well-studied sulfate-reducing bacteria was also included in the community model description [46]. The metabolic networks representing central carbon and energy metabolism for each guild were then constructed and three different modeling approaches based on the elementary mode analysis were employed to elucidate sustainable physiological properties of this community [46]. Here, we focus only on daylight metabolism (for which more experimental data is available) to assess the efficacy of OptCom in describing carbon and energy flows and the biomass ratio between guilds.

#### Analysis of the daylight metabolism

**Figure 4:**
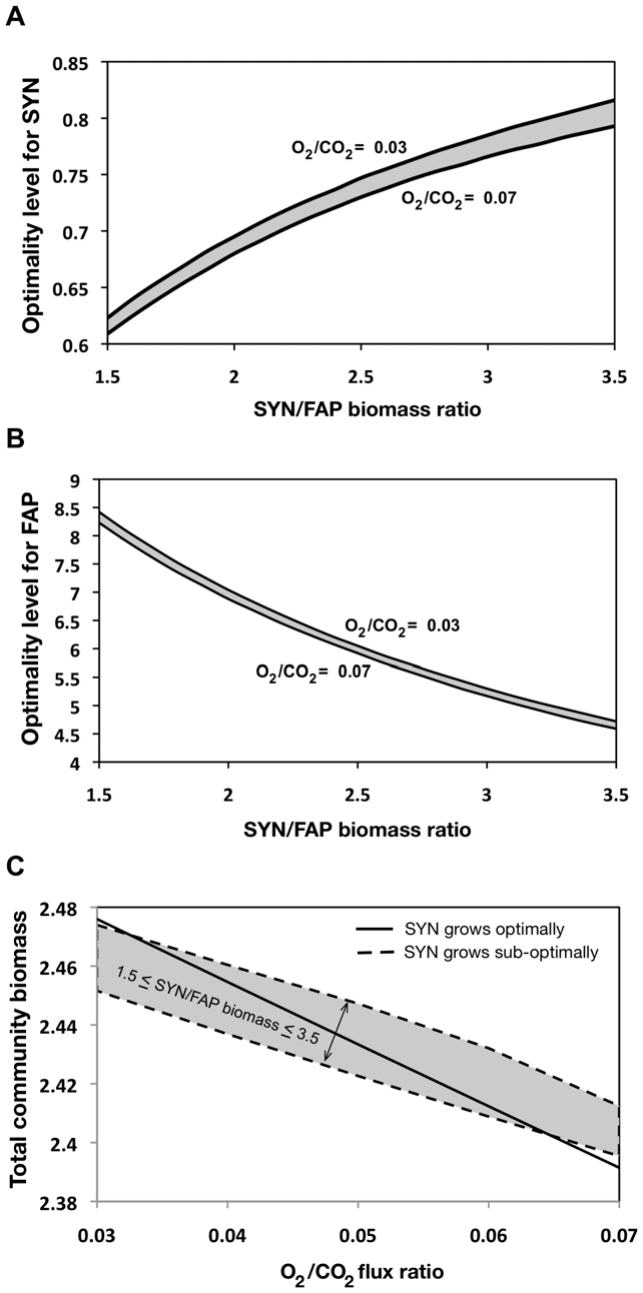
Optimality levels for the SYN and FAP guilds and their effect on the total community biomass. Optimality levels for (A) SYN and (B) FAP as a function of the SYN/FAP biomass ratio across different values of the O_2_/CO_2_ flux ratio (C) Comparison of the predicted total community biomass (1/h) for the case when SYN grows sub-optimally and when it grows optimally. Note that, to compute the total community biomass when SYN grows optimally only O_2_/CO_2_ flux ratio was fixed at values in the experimentally determined range (i.e., 0.03 to 0.07), whereas for all other cases, in addition to O_2_/CO_2_ flux ratio, SYN/FAP biomass ratio was also fixed at values measured experimentally (i.e., 1.5 to 3.5). Lower and upper dashed lines in (C) represent the maximum and minimum predicted community biomass (when SYN grows sub-optimally) across various SYN/FAP biomass ratios.

The relative abundance of various species in a microbial community (i.e., composition) is of significant ecological importance. The ratio of cyanobacterial (SYN) to FAP biovolumes in a Mushroom Spring mat was determined experimentally to be 1.6∶1 [67]. It was assumed that biomass formation rates and biovolume of species in the community are directly related [46]. In another study the biomass ratio in the top 1 mm of Octopus and Mushroom Spring mats was estimated to range from 1.5∶1 to 3.5∶1 based on the relative abundances of metagenomic reads [46]. We used OptCom to model this community postulating that each guild strives to maximize its biomass and examined if the biomass ratio of SYN/FAP can be correctly predicted. We chose as the outer problem objective function to maximize the total community biomass (i.e., SYN biomass+FAP biomass+SRB biomass). During the day O_2_ competes with CO_2_ for the rubisco active site, leading to production of glycolate (O_2_+ribulose−5−P+ATP→glycolate+triose phophate+ADP) instead of additional reduced carbon (CO_2_+ribulose−5−P+ATP→2 triose phophate+ADP) [46]. The flux ratio of these two reactions (O_2_/CO_2_) was measured for the Octopus and Mushroom Spring microbial mats and reported to vary approximately between 0.03 and 0.07 [46], [68]. We incorporated this information into our modeling framework by fixing the flux ratio of these reactions at different values between 0.03 and 0.07 (using a constraint in the inner problem of SYN). Lower and upper bounds on all reactions (except for the uptake and export fluxes of the shared metabolites) were taken from [46]. Under these conditions, the SYN/FAP biomass ratio was predicted to range from 7.94 (for O_2_/CO_2_ flux ratio = 0.07) to 20.26 (O_2_/CO_2_ flux ratio = 0.03), which are significantly higher than the experimentally determined values of 1.5 to 3.5. This suggests that the reason for the discrepancy in prediction may be that the SYN guild does not maximize its biomass. Therefore, we decided to test this hypothesis by using the descriptive mode of the OptCom procedure (see Figure 1B) and establish the optimality level of SYN and other members of this community. To this end, we added a constraint to the outer problem to fix the SYN/FAP biomass ratio at different values in the experimentally observed range (1.5 to 3.5). The objective function of the outer problem was assumed to be maximization of the total community biomass. We determined the optimality levels across different values of SYN/FAP biomass and O_2_/CO_2_ flux ratios in their experimentally determined ranges (see Figure 4). OptCom finds that the observed SYN/FAP biomass ratios are consistent with SYN guild growing sub-optimally at 61–82% of its community-specific maximum with lower values corresponding to higher O_2_/CO_2_ flux ratios (see Figure 4A). On the other hand, FAP guild appears to benefit from this sub-optimal behavior of SYN by growing at rates, which are approximately 4.5 to 8.5 times higher than its community-specific maximum (see Figure 4B).

SYN grows sub-optimally in this community to benefit other community members (e.g., FAP) and optimize a community-level fitness criterion (e.g., maximize the total community biomass). We investigated the effect of sub-optimal growth of the SYN guild on the total community biomass production across different values of SYN/FAP biomass and O_2_/CO_2_ flux ratios (see Figure 4C). As illustrated in Figure 4C, at higher O_2_/CO_2_ flux ratios, the total community biomass is higher compared to the case when SYN grows optimally. The metabolic reason for this lower growth of SYN is that fixing more carbon (manifested by 3–7 times more predicted glycolate and acetate production) to supply other guilds and increase the overall community biomass imposes extra energy demands on the SYN guild. In contrast, for low O_2_/CO_2_ flux ratios the maximum community biomass when SYN grows sub-optimally is lower compared with when it grows optimally (i.e., both dashed lines lie below the solid line in Figure 4C). A possible reason for this discrepancy is that the experimental measurements for SYN/FAP biomass ratio were performed when the O_2_/CO_2_ flux ratio was high. This could also be a consequence of the experimental underestimation of glycolate production due to consumption of radio-labeled photosynthate during incubation as stated in [46]. Alternatively, SYN may grow sub-optimally so that it can divert some resources towards polysaccharide production to fuel night-time maintenance energy and morning nitrogen fixation. This is another type of a cooperative behavior by SYN.

Notably, two different cases were considered by Taffs *et al* [46] using the elementary modes and compartmentalized approach: a selfish criterion where each guild attempts to maximize its own biomass and an altruistic criterion where the guilds strive to maximize the total community biomass. It was concluded that predictions using the first criterion are in better agreement with experimental data. OptCom, on the other hand reveals that a trade-off between these two criteria appears to be driving the metabolism in this community. While some guilds strive to maximize their own growth, others (e.g., SYN) grow sub-optimally to maximize the biomass of entire community or benefit the nighttime metabolism, or a combination of both, depending on O_2_/CO_2_ flux ratio and environmental conditions.

### Elucidating trophic and electron accepting interactions in sub-surface anaerobic environments

In a recent study, Miller *et al* [47] established a model microbial community to better understand the trophic interactions in sub-surface anaerobic environments. This community was composed of three species including *Clostridium cellulolyticum*, *Desulfovibrio vulgaris* Hildenborough, and *Geobacter sulfurreducens*. Cellobiose was provided as the sole carbon and energy source for *C. cellulolyticum* whereas the growth of *D. vulgaris* and *G. sulfurreducens* were dependent on the fermentation by-products produced by *C. cellulolyticum*. *D. vulgaris* and *G. sulfurreducens* were supplemented with sulfate and fumarate, respectively, as electron-acceptors to avoid electron acceptor competition [47]. The experimental measurements for the biomass composition of the community showed that, as expected, *C. cellulolyticum* was the dominant member in the co-culture and confirmed the presence of *D. vulgaris* and *G. sulfurreducens*. It was, however, not possible to quantify experimentally the flow of shared metabolites among the community members as their concentrations were below the detection limits. Therefore, the authors proposed an approximate model of the carbon and electron flow based on some measurements of the three species community at steady-state, pure culture chemostat experiments and data from the literature [47].

Here, we model this microbial community by making use of the corresponding bacterial metabolic models and employ OptCom to elucidate the inter-species interactions. The metabolic models of *C. cellulolyticum* (i.e., *i*FS431) and *G. sulfurreducens* were reconstructed by Salimi *et al* [33] and Mahadevan *et al* [69], respectively. A basic metabolic model of *D. vulgaris* containing 86 reactions was introduced by Stolyar et al [19], however, this model had only a compact representation of the central metabolism. For example, the model was not able to support growth in the presence of acetate or ethanol as the sole carbon source. Therefore, we expanded this model by adding new reactions from a first draft reconstructed model in the Model Seed [70] and the KEGG database [71] using the GrowMatch procedure [50] (see Text S1 for details). The updated model of *D. vulgaris* consists of 145 reactions and is capable of supporting growth on acetate as well as ethanol. This model is available in the supplementary material (Table S1).

#### Fumarate consumption by *G. sulfurreducens*

FBA simulations showed that the metabolic model for G. sulfurreducens [69] is not able to capture the experimental observation that the amount of fumarate consumed is higher than the amount of succinate produced. In addition, the model predicts that no malate is produced under the examined conditions. An inspection of the metabolic model of G. sulfurreducens revealed that the only included uptake pathway for fumarate is through mutual dicarboxylic acid transporter (fumarate[e]+succinate[c]↔fumarate[c]+succinate[e]) implying that the amount of succinate produced must be equal to the amount of fumarate consumed. Interestingly, in support of the observations by Miller et al [47], a recent study [72] has confirmed that the fumarate consumption rate by G. sulfurreducens is higher than the succinate production rate and demonstrated using ^13^C-based metabolic flux analysis that fumarate can be used as an additional carbon source through the TCA cycle where it is converted to malate by fumarase, and oxaloacetate via malate dehydrogenase. These findings suggest that the dcu gene family (responsible for the uptake of dicarboxylates such as fumarate) in G. sulfurreducens may have a dual function, i.e., they can act both mutually (with exchange of another compound such as succinate) or independently (i.e., protonated), similarly to those in E. coli [73]. This was verified by performing a bi-directional BLAST analysis that revealed high sequence similarity between the dcu gene families in G. sulfurreducens and E. coli. It is worth noting that addition of an alternative succinate transporter to the model could also have been another way of explaining the experimental data, however this hypothesis was not supported by the BLAST analysis. Therefore, in the absence of any other experimental data, we decided to add a protonated transport reaction for fumarate to the model. In our simulations we restricted the flux of this reaction to 15.5% of the fumarate transfer by dicaboxylic acid transporter based on the metabolic flux data under electron acceptor limited conditions [72].

#### Uncovering the inter-species metabolite transfers in the community

**Figure 5:**
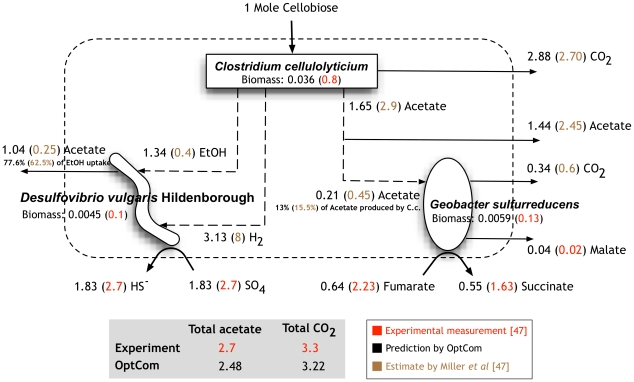
Comparison of the predicted fluxes by OptCom with estimates in the proposed model of [**47**]. The total predicted acetate and CO_2_ production rates by OptCom are in good agreement with experimental measurements by Miller *et al* [47]. Note that it was not possible to determine experimentally how much of the total acetate or CO_2_ available in the supernatant of the bioreactor is produced by which microorganism (the values provided by Miller *et al* [47] for the acetate and CO_2_ production by each species as well as all inter-organism flow rates are estimates and not experimental measurements). The values associated with the biomass of each microorganism represent fluxes (1/h) for OptCom predictions and concentrations (M) for experimental measurements [47].

While the relative molar abundance of each species was measured experimentally by Miller et al [47], the metabolite flows across community members were untraceable. We thus chose to use OptCom to gain insight into inter-species metabolite trafficking. To this end, we employed the descriptive mode of OptCom (see Figure 1B) first to establish the optimality levels of species participating in this community, by fixing the biomass composition of the community at the values obtained experimentally by adding constraints to the outer problem. The objective function of the outer problem was maximization of the total community biomass. Descriptive OptCom revealed that the experimentally determined biomass composition in this community was consistent with optimal growth for all microorganisms (i.e., optimality level of one for all species involved). Upon verifying that biomass maximization was driving metabolism in this community, we used OptCom to make predictions about inter-organism flow rates with a basis of 1 mole/gDW.hr of cellobiose uptake by C. cellulolyticum so that we can directly compare our results with the estimates in Miller et al [47]. The lower bound and upper bounds on all reactions (except for the uptake and export fluxes of the shared metabolites) were taken from the publications of the respective metabolic models [19], [33], [69]. Because D. vulgaris has a much more efficient enzymatic process for hydrogen consumption than G. sulfurreducens, we initially allowed G. sulfurredcens to take up only a small portion (between 1 to 10%) of the total hydrogen produced by C. cellulolyticum. However, the total predicted acetate and CO_2_ accumulation in the extracellular environment deviated significantly from the experimental observations by Miller et al [47]. Therefore, we decided to perform the remaining simulations assuming that D. vulgaris consumes all hydrogen produced by C. cellulolyticum (even though this may not be the only way of reconciling model predictions and the experimental data). OptCom found that under these conditions 1 mol/gDW.hr of cellobiose leads to 2.48 moles/gDW.hr of acetate and 3.22 moles/gDW.hr of CO_2_ in the extracellular environment which agree well with 2.7 and 3.3 moles/gDW.hr of acetate and CO_2_, respectively, observed in the supernatant of the bioreactor (per mole of cellobiose) by Miller et al [47]. We note, however, that the predicted level of acetate production by C. cellulolyticum metabolic model (1.65 mol/gDW.hr) is lower than what was estimated in Miller's model (2.9 mol/gDW.hr). In general, however, the predicted allocation of metabolic resources to different members of the community by OptCom is in good agreements with estimations in Miller [47] (see Figure 5). For example, OptCom suggests that about 13% of the acetate produced by C. cellulolyticum is directed towards G. sulfurreducens, which is very close to the 15.5% value estimated in [47].

OptCom results also show that hydrogen and ethanol produced by *C. cellulolyticum* can be completely utilized by *D. vulgaris* to reduce sulfate to hydrogen sulfide. A rough estimate for the ratio of hydrogen to ethanol, which serve as electron donors for *D. vulgaris*, is given in by Miller *et al* [47] (H_2_/Ethanol = 20) based on the pure culture data under similar conditions. The simulations with OptCom using genome-scale metabolic models of the community members, however, indicate a much higher contribution of ethanol in inter-species electron transfer (H_2_/Ethanol = 2.34). We performed a flux variability analysis to see if this ratio can change under the examined condition, while maintaining the maximum community biomass, but no changes in this ratio were possible. This suggests that under the observed experimental condition, a H_2_/Ethanol ratio of 2.34 is needed to support the maximum growth for each species as well as for the community as a whole. While acetate serves as the only carbon substrate for both *G. sulfurreducens* and *D. vulgaris*, it was not possible to determine experimentally if *D. vulgaris* directly uses the available acetate in the medium released by *C. cellulolyticum* or it derives acetate from ethanol. OptCom results support the latter scenario (see Figure 5). This is more likely to happen because acetate is already available internally to *D. vulgaris* from the cytosolic oxidation of ethanol. OptCom also identifies that 77.6% of the converted ethanol to acetate is secreted to the medium by *D. vulgaris*, while the rest is incorporated into biomass (see Figure 5). This is in good agreement with the estimate by Miller *et al* [47] suggesting that *D. vulgaris* does not consume any acetate produced by *C. cellulolyticum* and that it exports 62.5% of the assimilated ethanol to the medium as acetate. Elucidation of the metabolic interactions among the members of this community was achieved by OptCom after verifying that all species appear to grow optimally based on the *in vivo* observations for the community biomass composition.

#### Addition of a new member to the microbial community

As mentioned earlier, 2.48 moles/gDW.hr of acetate was predicted to be available in the extracellular environment (per mole of cellobiose consumed) which could be utilized by other trophic anaerobic bacteria [47]. Therefore, an acetate utilizing methanogen such as Methanosarcina species, which are known to be avid consumers of acetate, can be envisioned as an additional member of this community. We chose Methanosarcina barkeri for this analysis as its metabolic model has been reconstructed by Feist et al [74]. Another inner problem was added to the OptCom to account for addition of M. barkeri to this community. Consistent with other community members the objective function for this inner problem was to maximize the biomass flux of M. barkeri, whereas the objective function of the outer problem was to maximize the total community biomass. The acetate uptake rates by G. sulfurreducens and D. vulgaris were fixed at the values obtained by OptCom for the tri-culture. D. vulgaris and M. barkeri were suggested to compete in anoxic environments for hydrogen [75], however, we assumed that all H_2_ produced by C. cellulolyticum is consumed by D. vulgaris, as it has been reported to have much more favorable kinetic parameters for H_2_ metabolism than methanogens [76], [77], [78]. In addition, it was demonstrated that Methanosarcina species can not only consume but also produce hydrogen when growing on organic substrates such as acetate [79], [80]. Therefore, we allowed D. vulgaris to consume the hydrogen produced by M. barkeri (if any) in addition to that produced by C. cellulolyticum.

The biomass flux of *M. barkeri* is strongly dependent on the value of growth-associated maintenance (GAM), which was found to be a function of the proton translocation efficiency of the Ech hydrogenase reaction [74]. The range of GAM values for 0.2–2 protons translocated/2e^−^ that result in a growth yield consistent with *in vivo* observations was computed by Feist *et al* [74]. Here, we examined the variability in growth yields and relative abundance of *M. barkeri* in the tetra-culture community across different GAM values associated with 0.2–2 protons translocated/2e^−^. This analysis showed that *M. barkeri* is capable of consuming the entire 2.48 moles of acetate produced by *C. cellulolyticum* and *D. vulgari*. Depending on the GAM value and the proton translocation efficiency, *M. barkeri* was predicted to constitute 2.5 to 10.4% of the total community biomass (assuming that the biomass fluxes are proportional directly with the abundance levels of species in the community) with the other three members growing at rates similar to the ones obtained for the tri-culture. *C. cellulolyticum* still dominates the co-culture as before with biomass fractions ranging from 69.6 to 75.7% (depending on *M. barkeri*'s biomass flux). The methane evolution rate by *M. barkeri* was predicted by OptCom to range from 2.36 to 2.45 moles/gDM.hr. It is important to note that previous studies have reported that the internal carbon and electron flow of *M. barkeri* could be altered by *D. vulgaris* in a co-culture grown on an organic substrate such as acetate, [81]: It was suggested that *D. vulgaris* strives to keep the partial pressure of hydrogen low enough to shift the catabolic redox system of methanogen so that more H_2_ is produced by *M. barkeri* (compared to pure cultures) and more acetate is oxidized to CO_2_ instead of methane [81]. Even though we allowed *D. vulgaris* to take up all hydrogen produced by *M. barkeri* (in addition to that produced by *C. cellulolyticum*), no such shift in methanogenesis was observed for the tetra-culture according to the OptCom predictions. A possible reason might be that enough hydrogen (as well as ethanol) is already available to *D. vulgaris* from *C. cellulolyticum*, obviating the need to alter methanogenesis in order to gain the reducing equivalents. This hypothesis is supported by the experimental observation that if excess H_2_ is added to the co-culture of *M. barkeri* and *D. vulgaris*, it is completely consumed by *D. vulgaris* and the acetate catabolism by *M. barkeri* is no longer affected [81].

Even though 3.22 moles/gDW.hr of CO_2_ produced by *C. cellulolyticum* and *G. sulfurreducens* is available in the medium, OptCom predicts that it remains completely unused in the tetra-culture. This was expected as growth of *M. barkeri* on CO_2_ relies on presence of hydrogen, which we assumed that it was consumed completely by *D. valgaris*. In order to examine if *M. barkeri* is indeed capable of utilizing the available CO_2_ as a carbon source (in addition to acetate), we temporarily allowed *M. barkeri* to take up the hydrogen produced by *C. cellulolyticum*. For this case, OptCom revealed that if the entire hydrogen produced by *C. cellulolyticum* is available to *M. barkeri*, it can support growth on CO_2_ only for proton translocation efficiencies of less than one/2e^−^. Notably, for proton translocation efficiencies of more than one, even though no CO_2_ is assimilated by *M. barkeri*, OptCom shows that the availability of hydrogen will lead to an increase in the methane production by about 26–28%.

## Discussion

Here, we introduced OptCom, a comprehensive computational framework for the flux balance analysis of microbial communities using genome-scale metabolic models. We demonstrated that OptCom can be used for assessing the optimality level of growth for different members in a microbial community (i.e., Descriptive mode) and subsequently making predictions regarding metabolic trafficking (i.e., Predictive mode) given the identified optimality levels. Unlike earlier FBA-based modeling approaches that rely on a single objective function to describe the entire community [19], [30] or separate FBA problems for each microorganism [25], [27], [31], [33], OptCom integrates both species- and community-level fitness criteria into a multi-level/objective framework. This multi-level description allows for properly quantifying the trade-offs between selfish and altruistic driving forces in a microbial ecosystem. Species and community level fitness functions are quantified by maximizing the biomass formation for the respective entity. We note, however, that the physiology of microbial communities is highly context and environment dependent and a universal community-specific fitness criterion does not exist. Studies similar to those conducted for mono-cultures that examine and compare various presumed hypotheses on cellular objective function [82], [83], [84], [85], [86], [87] or algorithms that identify/test a relevant objective function using experimental flux data [88], [89] are needed in the context of multi-species systems.

An important goal of studying natural and synthetic microbial communities is their targeted manipulation towards important biotechnological goals (e.g., cellulose degradation, ethanol production, etc.). This has motivated researchers to construct simple synthetic microbial ecosystems, which are amenable to genetic and engineering interventions, for biotechnology- and bioenergy-related applications. As an example, Bizukojc *et al* [22], have proposed a co-culture composed of *Clostridium butyricum* and *Methanosarcina mazei* to relieve the inhibition of fermentation products and increase production of 1,3-propanediol (PDO) by *Clostridium butyricum*. Mixed cultures have been also established for overproduction of polyhydroxyalkanoates (PHA) [90], [91] and ethanol [92], [93], [94], [95], [96]. For example, *Clostridium thermocellum*, which is used for ethanol production, has been found to be capable of utilizing hexoses, but not pentose sugars generated from breakdown of cellulose and hemicellulose [96]. Therefore, cultivation of *C. thermocellum* with other thermophilic anaerobic bacteria capable of utilizing hexoses as well as pentose to produce ethanol (e.g., *Clostridium thermosaccharolyticum* and *Thermoanaerobacter ethanolicus*) has been previously examined *in vivo* [92], [93], [94], [95], [96]. The multi-objective and multi-level structure of the OptCom procedure, introduced here, can help assess the metabolic capabilities of such synthetic ecosystems. Taking a step further, OptCom can be readily modified to identify the minimal number of direct interventions (i.e., knock-up/down/outs) to the community leading to the elevated production of a desired compound (e.g., by considering the overproduction of desired compound as the outer problem objective function), thus extending the applicability of strain design tools such as OptKnock [48], OptStrain [49], OptReg [97] and OptForce [98]. It is worth noting that a key bottleneck to the modeling and analysis of microbial communities is the paucity of genome-scale models for all participants in a complex microbial community. Overcoming this barrier would require the development of high-throughput metabolic reconstruction tools such as the Model Seed [70] resource. Given that microbial communities change with time (e.g., day/night cycle) and also location (e.g., nutrient gradients), approaches that would be able to capture temporal and spatial varying inter-species metabolic interactions are needed. For example, the separate FBA problems for each individual species in the dynamic flux balance analysis methods of Zhuang *et al* [31] and Tzamali *et al* [25], [27] can be integrated with OptCom to account for inter-species interactions and community-level fitness driving forces within each time interval.

## Supporting Information

Table S1 Updated model of *D. vulgaris* and details of the model corrections.
(XLSX)

Text S1 Optimization formulation and solution procedure for OptCom and details of the update procedure for the metabolic model of *D. vulgaris*.
(PDF)
