# Supplementary material for: OptCom: A Multi-Level Optimization Framework for the Metabolic Modeling and Analysis of Microbial Communities
Source: PLoS Comput Biol. 2012 Feb 2;8(2):e1002363. doi: 10.1371/journal.pcbi.1002363 (PMC3271020; doi:10.1371/journal.pcbi.1002363)
Supplement: Text S1 — Optimization formulation and solution procedure for OptCom and details of the update procedure for the metabolic model of D. vulgaris. (PDF) [file pcbi.1002363.s002.pdf]

Text S1

OptCom: A Multi-Level Optimization Framework for the Metabolic Modeling and Analysis of Microbial Communities

Ali R. Zomorrodi and Costas D. Maranas

Department of Chemical Engineering, The Pennsylvania State University, University Park, PA 16802, USA

Mathematical Description of OptCom

Mathematical description of OptCom for the flux balance analysis of microbial communities requires definition of the following sets:

$\boldsymbol{K}$ = {k | k = 1,2,…,K} = Set of microorganisms (or guilds) present in the community

$\boldsymbol{J^k}$ = {j | j = 1,2,… N^k} = Set of reactions in microorganism (or guild) k

$\boldsymbol{I^k}$ = {i | i = 1,2,…, M^k} = Set of metabolites in microorganism (or guild) k

$\boldsymbol{I}^k_{uptake}$ and $\boldsymbol{I}^k_{export}$ = Set of metabolites that are exported or taken up by microorganism (or guild) k and are shared (exchanged) with other community members ($\boldsymbol{I}^k_{uptake}$, $\boldsymbol{I}^k_{export} \subset \boldsymbol{I}^k$).

$\boldsymbol{I}_{shared}$ = Set of the shared metabolites that are present in the extra-cellular environment and are shared (exchanged) among the community members. Note that for each metabolite in this set, there exists at least one corresponding metabolite in $\boldsymbol{I}^k_{uptake}$ or $\boldsymbol{I}^k_{export}$ for some $k \in \boldsymbol{K}$.

We also, define the following variables and parameters using these sets:

Variables:
v^k_j = Flux of reaction j in microorganism (or guild) k.
e^k_i = Export flux of metabolite $i \in \boldsymbol{I}^k_{export}$ by microorganism (or guild) k $\left(e^k_i \in \left\{v^k_j\right\}\right)$.
u^k_i = Uptake flux of metabolite $i \in \boldsymbol{I}^k_{uptake}$ by microorganism (or guild) k $\left(u^k_i \in \left\{v^k_j\right\}\right)$.
e^C_i = Export flux of the shared metabolite $i \in \boldsymbol{I}_{shared}$ by the community.

u_i^c = Uptake flux of the shared metabolite $i \in \boldsymbol{I}_{shared}$ by the community.

Parameters:
S_{ij}^k = Stoichiometric coefficient of metabolite $i \in \boldsymbol{I}^k$ in reaction $j \in \boldsymbol{J}^k$.
LB_j^k = Lower bound on reaction $j \in \boldsymbol{J}^k$ in microorganism (or guild) *k*.
UB_j^k = Upper bound on reaction $j \in \boldsymbol{J}^k$ in microorganism (or guild) *k*.

OptCom is a multi-level and multi-objective optimization problem, which can be represented as following:

$$\begin{aligned}
&\mathit{Maximize/Minimize} \quad z = \text{Community-level objective} \qquad \text{[OptCom]}\\
&\text{subject to}\\
&\left[\begin{array}{lll}
\underset{v_j^k}{\mathit{Maximize}} & v_{biomass}^k & \\
\text{subject to} & & \\
 & \sum_{j \in \boldsymbol{J}^k} S_{ij}^k . v_j^k = 0 & \forall \ i \in \boldsymbol{I}^k \quad (1)\\
 & LB_j^k \le v_j^k \le UB_j^k & \forall \ j \in \boldsymbol{J}^k \quad (2)\\
 & u_i^k = uval_i^k & \forall \ i \in \boldsymbol{I}_{uptake}^k \quad (3)\\
 & e_i^k = eval_i^k & \forall \ i \in \boldsymbol{I}_{export}^k \quad (4)
\end{array}\right] \quad \forall \ k \in \boldsymbol{K}\\
&\sum_k uval_i^k + e_i^c = \sum_k eval_i^k + u_i^c \qquad \forall \ i \in \boldsymbol{I}_{shared} \quad (5)\\
&uval_i^k, eval_i^k, e_i^c, u_i^c \ge 0 \qquad \forall \ i \in \boldsymbol{I}_{shared}\ , \ k \in \boldsymbol{K}
\end{aligned}$$

Constraint (1) in the inner problems represents the steady-state mass balance for each microorganism (or guild) *k*. Constraint (2) imposes a lower and upper bound for each flux. Constraints (3) and (4) fix the uptake or export flux of a shared metabolite *i* at the values $uval_i^k$ and $eval_i^k$, respectively, which are imposed by the outer problem. This means that $uval_i^k$ and $eval_i^k$ serve as variables for the outer problem, but act as parameters for the inner problems. Constraint (5) in the outer problem establishes a mass balance for each shared metabolite $i \in \boldsymbol{I}_{shared}$ present in the extra-cellular environment (shared metabolite pool), where the terms $\sum_k uval_i^k$ and $\sum_k eval_i^k$ represent the total uptake and export of the shared metabolite *i* by community members, respectively. This constraint is the key equation modeling the interactions and communications among participants of the community (through exchanging or sharing metabolites). Any other microorganism or community related conditions can be readily incorporated into the OptCom formulation through addition of appropriate constraints to the inner or outer problems, respectively. A pictorial representation of this formulation is given in Figure TS1.1.

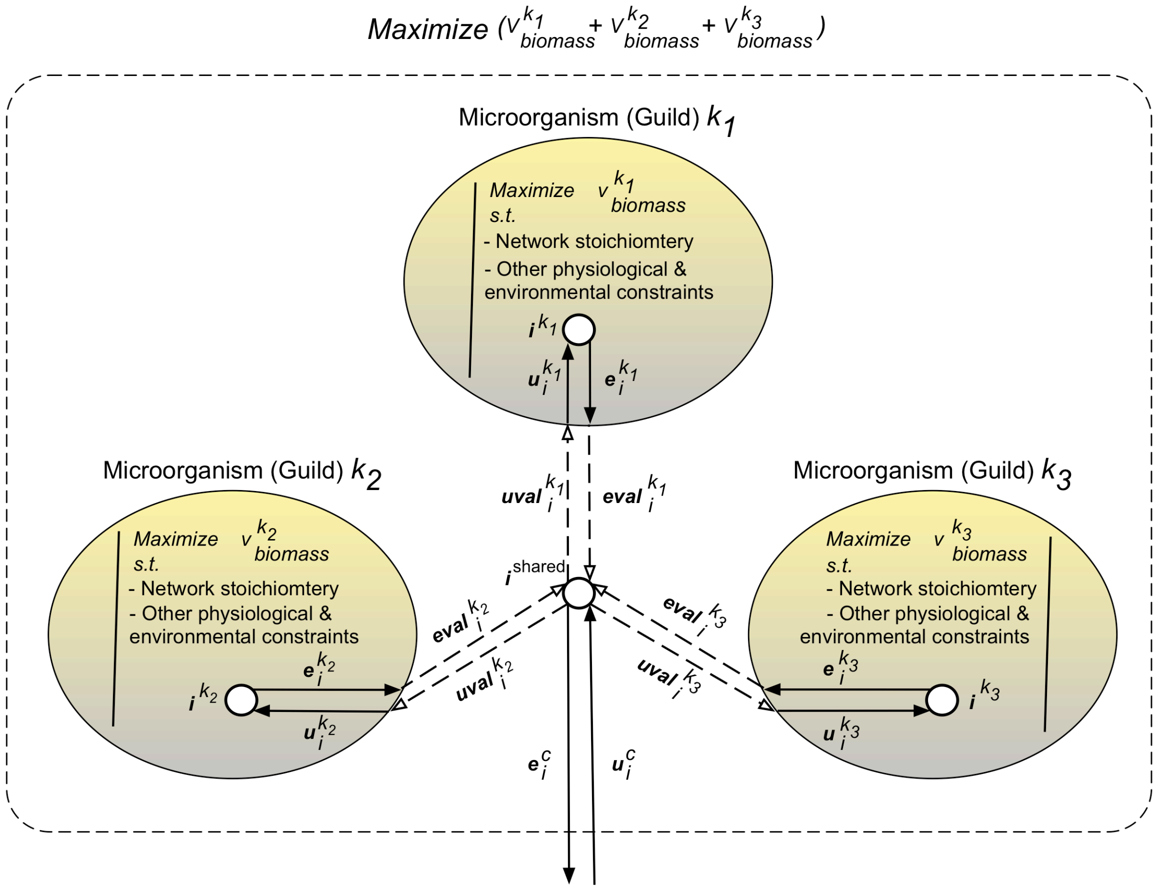

Figure TS1.1- Graphical representation of the variables and equations defined in the OptCom formulation for a sample community composed of three microorganisms (guilds). In this figure *i* is a typical metabolite that is exchanged (shared) among community members. The community-level objective function is assumed to be maximization of the total community biomass.

The multi-level structure of the OptCom implies that the inner problems are integrated in the outer stage so as a community-level objective function is primarily optimized (e.g., maximization of the total community biomass), while the fluxes are further constrained (by the inner problems) to maximize an individual-level criterion (i.e., growth). Solution methods of the bilevel problems from [1,2] are used to convert the multi-level program of OptCom to a bilinear optimization problem by adding the constraints of the dual of each inner problem and setting the objective functions for the primal and dual problems equal to one another:

$$\text{Maximize} \qquad z = \underset{\text{(e.g., total community biomass)}}{\text{Community - level objective}} \qquad \text{[OptCom]}$$

subject to

$$\sum_{j \in \boldsymbol{J}^k} S_{ij}^k . v_j^k = 0 \qquad \forall \quad i \in \boldsymbol{I}^k \; , \; k \in \boldsymbol{K} \qquad (1)$$

$$LB_j^k \le v_j^k \le UB_j^k \qquad \forall \quad j \in \boldsymbol{J}^k \; , \; k \in \boldsymbol{K} \qquad (2)$$

$$u_i^k = uval_i^k \qquad \forall\ i \in \boldsymbol{I}_{uptake}^{\boldsymbol{k}}\ ,\ k \in \boldsymbol{K} \tag{3}$$

$$e_i^k = eval_i^k \qquad \forall\ i \in \boldsymbol{I}_{export}^{\boldsymbol{k}}\ ,\ k \in \boldsymbol{K} \tag{4}$$

$$\sum_{i \in \boldsymbol{I}^k} S_{ij}^k.\lambda_i^k + \mu_j^k - \eta_j^k = 0 \qquad \forall\ j \in \boldsymbol{J}^k - \left(\left\{v_{biomass}^k\right\} \cup \left\{u_i^k \middle| i \in \boldsymbol{I}_{\boldsymbol{uptake}}^{\boldsymbol{k}}\right\} \cup \left\{e_i^k \middle|\ i \in \boldsymbol{I}_{\boldsymbol{export}}^{\boldsymbol{k}}\right\}\right)\ ,\ k \in \boldsymbol{K} \tag{6}$$

$$\sum_{i \in \boldsymbol{I}^k} S_{ij}^k.\lambda_i^k + \mu_j^k - \eta_j^k + \alpha_j^k + \beta_j^k = 0 \qquad \forall\ j \in \left(\left\{u_i^k \middle| i \in \boldsymbol{I}_{\boldsymbol{uptake}}^{\boldsymbol{k}}\right\} \cup \left\{e_i^k \middle|\ i \in \boldsymbol{I}_{\boldsymbol{export}}^{\boldsymbol{k}}\right\}\right)\ ,\ k \in \boldsymbol{K} \tag{7}$$

$$\sum_{i \in I^k} S_{i,biomass}^k.\lambda_i^k + \mu_{biomass}^k - \eta_{biomass}^k = 1 \tag{8}$$

$$v_{biomass}^k = \left(\sum_{j \in \mathbf{J}^\mathbf{k}} UB_j^k.\mu_j^k - \sum_{j \in \mathbf{J}^\mathbf{k}} LB_j^k.\eta_j^k + \sum_{j \in \left\{u_i^k \middle| i \in \mathbf{I}_{\mathbf{uptake}}^{\mathbf{k}}\right\}} uval_j^k.\alpha_j^k + \sum_{J \in \left\{e_i^k \middle|\ i \in \mathbf{I}_{\mathbf{export}}^{\mathbf{k}}\right\}} \mathbf{eval}_i^k.\beta_j^k \right) \quad \forall\ k \in \mathbf{K} \tag{9}$$

$$\sum_k uval_i^k + e_i^c = \sum_k eval_i^k + u_i^c \qquad \forall\ i \in \boldsymbol{I}_{shared} \tag{5}$$

$$uval_i^k, eval_i^k, e_i^c, u_i^c \geq 0 \qquad \forall\ i \in \boldsymbol{I}_{shared}\ ,\ k \in \boldsymbol{K}$$

$$\mu_j^k, \eta_j^k \geq 0 \qquad \forall\ j \in \boldsymbol{J}^{\boldsymbol{k}}\ ,\ k \in \boldsymbol{K}$$

$$\lambda_i^k, \alpha_j^k, \beta_j^k \in \boldsymbol{R} \qquad \forall\ i \in \boldsymbol{I}^k\ ,\ j \in \left\{u_i^k \middle| i \in \boldsymbol{I}_{\boldsymbol{uptake}}^{\boldsymbol{k}}\right\} \cup \left\{e_i^k \middle|\ i \in \boldsymbol{I}_{\boldsymbol{export}}^{\boldsymbol{k}}\right\}\ ,\ k \in \boldsymbol{K}$$

Here, λ_i^k, α_j^k, β_j^k (not restricted in sign), μ_j^k and η_j^k (non-negative) are the dual variables associated with the steady-state mass balance (constraint 1), uptake and export constraints (constraints 3 and 4) and right- and left-hand side inequalities in constraint (2), respectively. Equations (7) and (8) represent the dual constraints corresponding to uptake and export reactions for shared metabolites and the biomass reaction, respectively. Equation (6) is the dual constraint corresponding to all other reactions. Constraint (9) states that the objective functions of the primal and dual problems should be equal to achieve optimality (for inner problems). The bilinear formulation for OptCom is in general non-convex. It can be solved to optimality using the global optimization solver BARON [3]. Please note that problems with a few thousand bilinear terms in the constraints are typically solvable by BARON to global optimality. Given that the community members usually share only a limited number of metabolites the number of bilinear terms remain far below this limit for most cases. All examples presented in the manuscript were solved using this solver to achieve the global optimum. If an alternate nonlinear objective function for the inner problem such as minimization of the metabolic adjustment (MOMA) [4] deemed to be a better surrogate for cellular fitness, then instead of writing the dual constraints, one needs to write the Karush-Kuhn-Tucker (KKT) conditions for the inner problems (provided that they are convex).

Determining the optimality levels using Descriptive OptCom

In OptCom each species is assumed to maximize its own growth (i.e., in the inner problems). However, it happens quite often in microbial communities that individual microorganisms sacrifice in order to benefit other community member and improve an altruistic performance criterion. Therefore, individual species may grow at sub-optimal levels (e.g., 90% of the maximal growth rate) to benefit the entire population. This behavior can be identified and captured by OptCom through a modification of the

optimization structure presented above. The idea is to quantify the level of optimality of growth for each species in the community consistent with the set of available experimental data. To this end, we introduce a new metric called optimality level for each species k in the community (c^k) that captures the deviation of individual species growth from their optimal behavior. Optimality levels can be determined upon incorporating all available experimental data related to the whole community (e.g., the biomass composition of the community) as constraints in the outer problem and any data related to individual microorganisms as constraints in the respective inner problems. This new mode of OptCom is called Descriptive OptCom and can be represented as following:

$$\textit{Maximize/Minimize} \quad z = \text{(Community - level objective)} \qquad \text{[Descriptive OptCom]}$$

$$\textit{subject to}$$

$$\left[\begin{array}{lll} \underset{v_j^k}{\textit{Maximize}} & v_{biomass}^{k} & \\ \textit{subject to} & & \\ & \sum_{j \in \boldsymbol{J}^k} S_{ij}^k . v_j^k = 0 \quad \forall\ i \in \boldsymbol{I}^k & (1) \\ & LB_j^k \le v_j^k \le UB_j^k \quad \forall\ j \in \boldsymbol{J}^k & (2) \\ & u_i^k = uval_i^k \quad \forall\ i \in \boldsymbol{I}_{\text{uptake}}^k & (3) \\ & e_i^k = eval_i^k \quad \forall\ i \in \boldsymbol{I}_{\text{export}}^k & (4) \\ & v_{biomass}^{k} \le c^k . \text{vopt}_{\text{biomass}}^{\text{k}} & (10) \\ & \text{Constraints describing experimental} & \\ & \text{data for microorganism } k & (11) \end{array}\right] \quad \forall\ k \in \boldsymbol{K}$$

$$\sum_k uval_i^k + e_i^c = \sum_k eval_i^k + u_i^c \qquad \forall\ i \in \boldsymbol{I}_{\text{shared}} \quad (5)$$

Constraints describing experimental data for the whole community (12)

$$uval_i^k, eval_i^k, e_i^c, u_i^c, c^k \ge 0 \qquad \forall\ i \in \boldsymbol{I}_{\text{shared}}\ ,\ \ k \in \boldsymbol{K}$$

Constraint (10) allows the biomass flux for each microorganism (or guild) to be lower ($c^k < 1$), equal ($c^k = 1$), or higher ($c^k > 1$) than its optimum ($\text{vopt}_{\text{biomass}}^{\text{k}}$). Note that $\text{vopt}_{\text{biomass}}^{\text{k}}$ for each species is community-specific and is computed in the context of all microorganisms strive to maximize their own growth (using the [OptCom] formulation described in the previous section). The optimality level for each microorganism is a variable and is determined by solving the Descriptive OptCom. An optimality level of less than one for a microorganism k implies that it grows sub-optimally at a rate equal to $100c^k$ % of the maximum ($\text{vopt}_{\text{biomass}}^{\text{k}}$) to optimize a community-level fitness criterion while matching experimental observations. Alternatively, an optimality level of one implies that microorganism k grows exactly optimally at a rate equal to $\text{vopt}_{\text{biomass}}^{\text{k}}$

whereas a value greater than one indicates that it achieves a higher biomass production level than the community-specific maximum (i.e., super-optimality) by depleting resources from one or more other community members. It is worth noting that super-optimality for a species is achievable only at the cost of at least one other community member growing sub-optimally. Note that the presence of inner problems in Descriptive OptCom allows each species to still optimize its fitness (i.e., maximize its own biomass) as much as possible considering the available experimental data. However, once the optimality levels are determined (i.e., when the RHS of equation 10 is a constant) the inner problems can be replaced with their respective constraints and the problem is simplified to a single-level optimization problem, which can be used to provide further predictions about the community.

Customizing OptCom for various types of interactions

Here we consider various types of interactions among members of a microbial community and show how the general form of the OptCom can be customized for each case. The symbiotic interactions among two (or more) populations can be divided into two major categories: (*i*) *positive interactions*, where, one or more populations benefit(s) from the association, and (*ii*) *negative interactions,* where, one or more population(s) are negatively affected. All of these interactions can be modeled by customizing the inter-organism flow constraints (i.e., constraint 5 in the OptCom formulation). In the following we present all formulations considering only two interacting populations, however, they can be easily extended to more than two populations using the general from of OptCom as a basis. A summary is provided in Table TS1.1.

Mutualism

In mutualism both partners benefit from each other and this association is vital for one or both of them. Mutualism can be further divided into the following two categories:

1) *Syntrophy*: is a mutualistic relationship between two microorganisms, which together degrade an otherwise indigestible organic substrate. Here, the metabolite produced by one microorganism has to be consumed by the other in order to stimulate the growth of both species (see the first example in the manuscript for more details). Assume microorganism k_1 produces a metabolite i, which inhibits its own growth but can be scavenged by its syntrophic partner k_2 (see Figure S1.2A). Constraint (5) can then be written as following:

$$uval_i^{k_2} = eval_i^{k_1}$$

This equation implies that the production and export rate of metabolite i by k_1 is completely dependent on its rate of uptake by k_2.

2) *Cross-talk*: where the growth of each microorganism is dependent on the product(s) of the other. An example of cross talk is two auxotrophic strains of the same

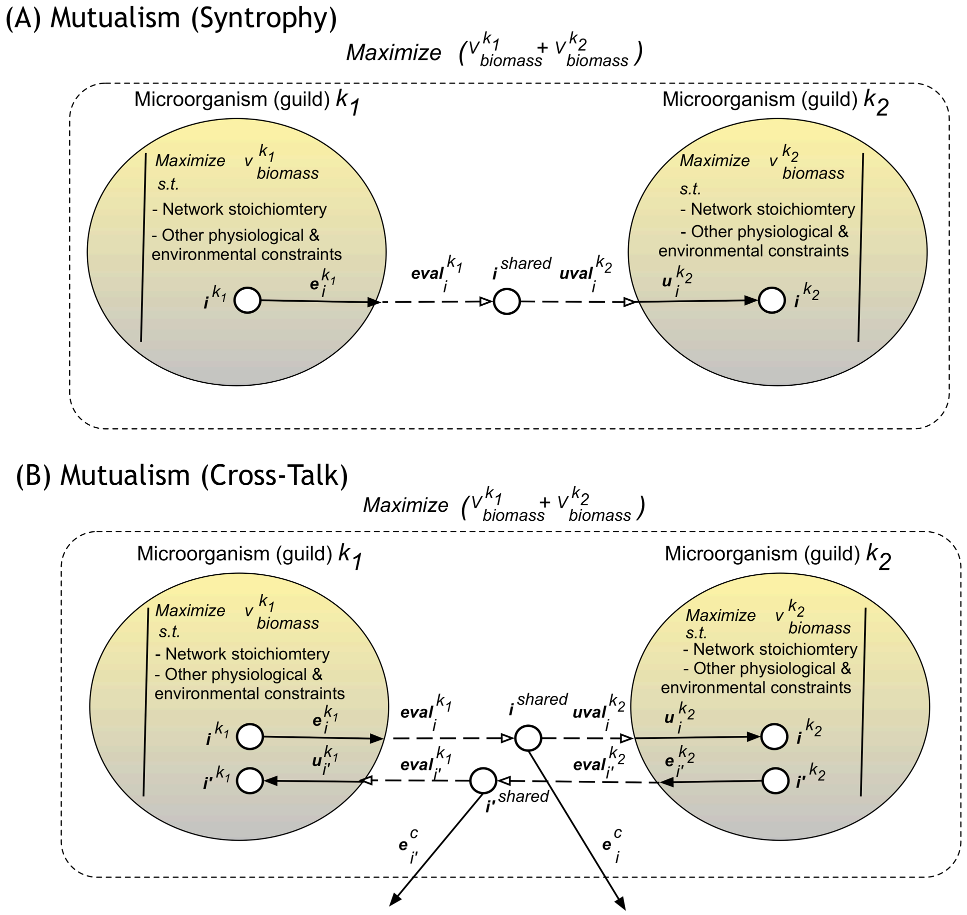

Figure TS1.2- Pictorial representation of the customized OptCom for mutualism. (A) Syntrophy, (B) Cross-talk.

species that grow on each other's produced compounds [5]. Let assume microorganisms k_1 and k_2 have such a relationship, where, microorganism k_2 takes up a metabolite i produced and exported by k_1 and microorganism k_1 grows on a compound i' produced and secreted by k_2 (see Figure TS1.2B). Constraint (5) for a mutualistic relationship can then be written as following:

$$uval_i^{k_2} + e_i^c = eval_i^{k_1}$$

$$uval_{i'}^{k_1} + e_{i'}^c = eval_{i'}^{k_2}$$

Synergism

Synergism is similar to mutualism in that both partners benefit from each other, but a synergistic association is not obligatory and each microorganism is able to survive in the absence of the other, This is because the metabolites produced by a partner is not essential for growth of the other, or otherwise there exists an external supply of that metabolite. These will make the growth of both microorganisms independent of one

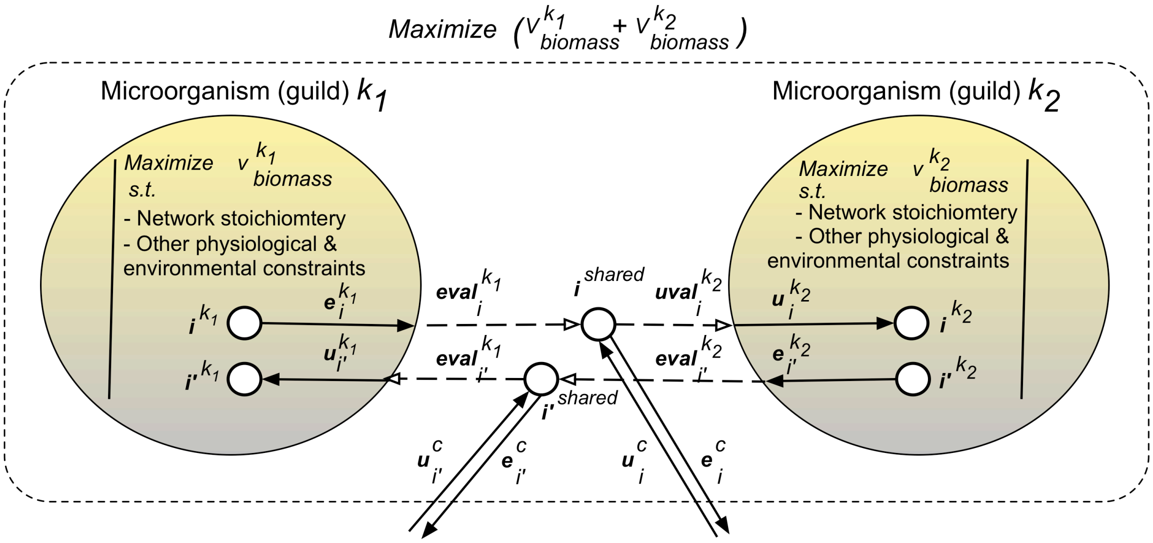

Figure TS1.3- Pictorial representation of the customized OptCom for synergism

another, however, they still benefit from each other. If population k_2 benefits from metabolite i produced by population k_1 and k_1 benefits from metabolite i' produced by k_2 (see Figure TS1.3) then constraint 5 can be written as following to represent this relationship:

$$uval_i^{k_2} + e_i^c = eval_i^{k_1} + u_i^c$$
$$uval_{i'}^{k_1} + e_{i'}^c = eval_{i'}^{k_2} + u_{i'}^c$$

Commensalism

Commensalism is a type of association where one of the microorganisms benefits from association, while the other remains unaffected (e.g., see [6]). This association is usually not obligatory for the two populations involved. Assume as an example, microorganism k_2 benefits from a metabolite i produced and exported by microorganism k_1 (see Figure TS1.4). Constraint (5) for this case can be written as following:

$$uval_i^{k_2} + e_i^c = eval_i^{k_1} + u_i^c$$

Note that since population k_1 is not affected by population k_2 in this case, one may remove the inner problem for k_1 and move all its constraints to the outer problem (i.e., OptCom is simplified to a bilevel optimization problem with a single inner problem). However, more caution should be exercised in this case when choosing the objective function of the outer problem as maximization of the total community biomass may cause population k_1 to grow sub-optimally in the interest of increasing sum of the biomass fluxes (i.e., community biomass). To avoid this, $v_{biomass}^{k_2}$ in the objective function of the outer problem should be replaced with $\varepsilon . v_{biomass}^{k_2}$ (ε is a small value), or alternatively a separate inner problem should be considered for k_1 (Figure T$1.4).

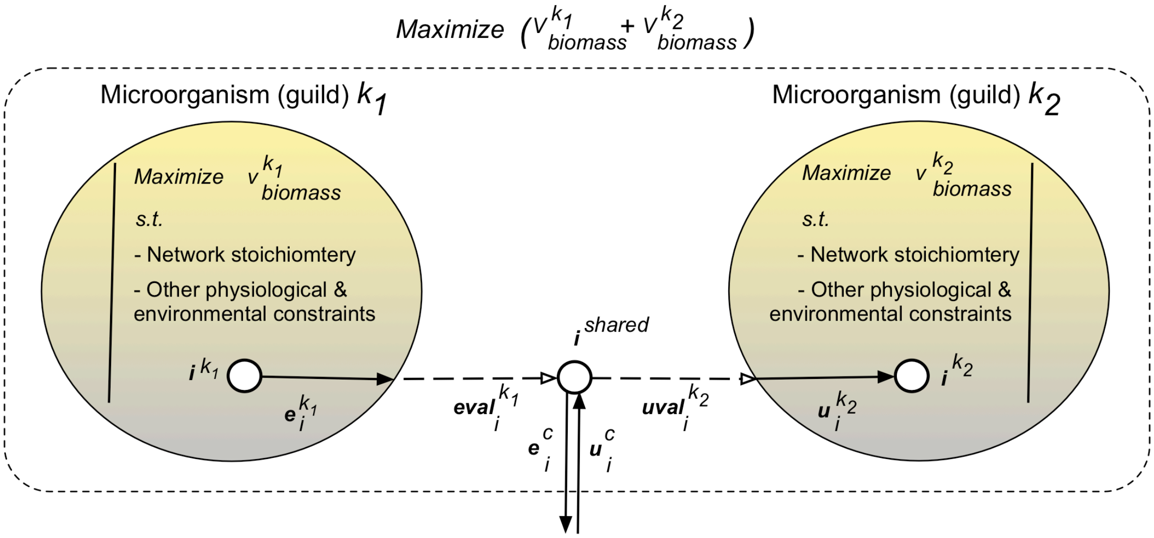

Figure TS1.4- Pictorial representation of the customized OptCom for commensalism

Parasitism

Parasitism is a type of negative association, which is advantageous to one of the populations but is harmful to the other (i.e., host). This negative effect may be destructive, where the host cell is completely destroyed in its later stages of development, or balanced, where demands from the host are in such a way that it is not destroyed and continue to live [7]. Assume microorganism k_1 acts as a host for microorganism k_2 through supplying metabolite i to it (see Figure TS1.5). Constraint (5) for this case can be written as following:

$$uval_i^{k_2} = eval_i^{k_1}$$

Since microorganism k_2 imposes an extra burden on the host (i.e., k_1) to produce the metabolite i, it will not be able to grow at its maximum rate. Therefore, an appropriate objective function for the outer problem is to maximize only the biomass flux of k_2. This implies that a higher growth rate for k_2 has a higher priority in this community. We note that if any experimental data is available about the composition of the biomass in such communities (i.e., where both species can co-exist), we can use the Descriptive OptCom to determine the optimality level of the host.

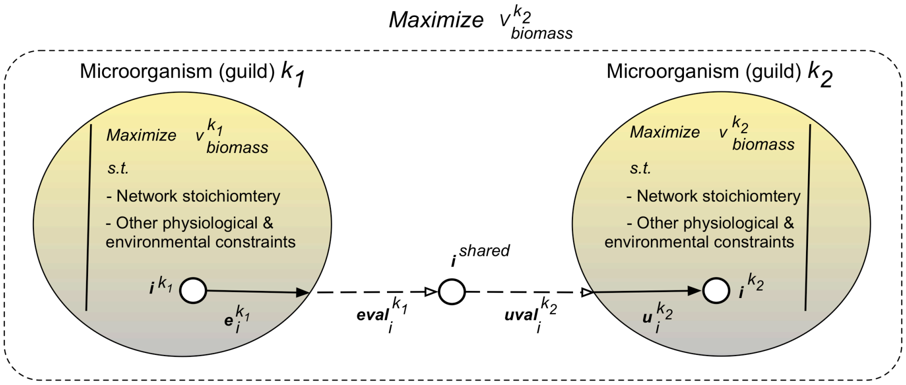

Figure TS1.5- Pictorial representation of the customized OptCom for parasitism.

Competition

Competition is a negative interaction between two (or more) microorganisms that may affect both populations in terms of their growth or survival. Usually, in these associations the microbial populations compete for limiting resources. The outcome of this competition may be the dominance of one population over the other (while they both continue to live) or complete removal of unsuccessful competitor(s) (e.g., see [8]). Assume populations k_1 and k_2 compete for a limiting substrate i (see Figure TS1.6). In this case the inter-organism flow constraint (constraint 5) can be adjusted as following to model the competition:

$$uval_i^{k_1} + uval_i^{k_2} = u_i^c$$

Note that when two or more microorganisms compete for a limiting resource they may act quite selfishly and a community-level criterion such as maximization of the total community biomass might not be biologically relevant anymore. Which microorganism will dominate at steady state in this selfish competing community is largely dependent on the ability of individual players to uptake the limiting substrate efficiently. If this efficiency information for each microorganism is available (e.g., from kinetic data) it can be incorporated into the OptCom formulation by assigning appropriate weights to $uval_i^{k_1}$ and $uval_i^{k_2}$ in the above equation. In this case maximizing the total community biomass can be a satisfactory objective function for the outer problem from a mathematical point of view. In the absence of this additional information, OptCom can be used only to explore the space of all possible physiological outcomes at steady state.

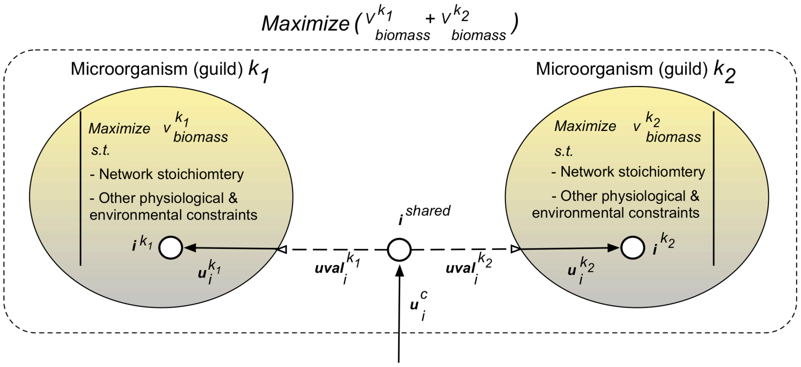

Figure TS1.6- Pictorial representation of the customized OptCom for competition.

Table TS1.1- A summary of different types of interactions in microbial communities and the way OptCom should be customized for each case. Here k_1 and k_2 refer to two interacting populations. In addition *i* and *i'* refer to metabolites produced by k_1 and k_2, respectively.

Type of interaction	***Description***	***Customized form of constraint (5)***
Mutualism (Syntrophy)	*Positive and obligatory*: Metabolite *i* produced by k_1 has to be consumed by k_2 to stimulate the growth of both species	$uval_i^{k_2} = eval_i^{k_1}$
Mutualism (Cross-talk)	*Positive and obligatory*: k_2 takes up metabolite *i* produced by k_1 and k_1 grows on compound *i'* produced by k_2.	$uval_i^{k_2} + e_i^c = eval_i^{k_1}$ $uval_{i'}^{k_1} + e_{i'}^c = eval_{i'}^{k_2}$
Synergism	*Positive but not obligatory*: k_2 benefits from metabolite *i* produced by k_1 and k_1 benefits from metabolite *i'* produced by k_2. Metabolites *i* and *i'* are not essential for growth of k_2 and k_1, respectively, or otherwise, they can be obtained from an external source.	$uval_i^{k_2} + e_i^c = eval_i^{k_1} + u_i^c$ $uval_{i'}^{k_1} + e_{i'}^c = eval_{i'}^{k_2} + u_{i'}^c$
Commensalism	*Positive but not obligatory*: k_2 benefits from metabolite *i* produced by k_1, however, k_1 is not affected by k_2. Metabolite *i* is not essential for growth of k_2, or otherwise, it can be obtained from an external source.	$uval_i^{k_2} + e_i^c = eval_i^{k_1} + u_i^c$ One may move the constraints for k_1 to the outer problem and use $v_{biomass}^{k_1} + \varepsilon . v_{biomass}^{k_2}$ as the objective function for the outer problem (ε is a small value).
Parasitism	*Negative*: k_1 acts as a host for k_2 through supplying metabolite *i* to it. k_1 is negatively affected, while k_2 benefits.	$uval_i^{k_2} = eval_i^{k_1}$ Maximize $v_{biomass}^{k_2}$ as the outer-problem objective function
Competition	*Negative*: k_1 and k_2 compete for a limiting substrate *i* in the medium	$uval_i^{k_1} + uval_i^{k_2} = u_i^c$ Assign appropriate weights to $uval_i^{k_1}$ and $uval_i^{k_2}$ according to the efficiencies of k_1 and k_2 for uptake of *i*.

Update procedure for the metabolic model of *Desulfovibrio vulgaris*

We used the input files of the FluxAnalyzer provided in the supplementary material of Stolyar *et al* [9] as the basis. This original model contains 86 reactions and 82 metabolites. We realized that a number of reactions in this model are not co-factor balanced and there are also other compounds missing from the reaction equations. Therefore, we investigated all reactions in the model manually and fixed the erroneous reactions through comparing the reaction equation with the corresponding equation (if available) in the KEGG database (http://www.genome.jp/kegg/) or the Model Seed (http://www.theseed.org/models/). This analysis resulted in correction of 62 reactions. However, neither the original model nor the corrected one could support growth in presence of acetate or ethanol.

We next queried the Model Seed to reconstruct a first-draft metabolic model for *D. vulgaris*. We found that this draft model could support growth only in the complete medium where all metabolites can be taken up. This was expected because the draft Seed model is only a preliminary reconstruction and requires at least ten additional curation steps [10] that should be done manually. Therefore, we decided to take advantage of both Stolyar's model, which was constructed and curated manually, and the draft Seed model through combining them. To this end, we first included all reactions that are present in the draft Seed model but are missing in the corrected Stolyar's model and examined if it can produce all required biomass precursors. We found that even in this case the model is not able to support growth in presence of any of the carbon sources mentioned above. Therefore, we employed the GrowMatch procedure [1] to identify additional reactions that need to be added to this integrated model from the KEGG database. This analysis led to identification of five new reactions that need to be added to the model. The presence of these reactions in the model was confirmed by performing a bi-directional protein-protein BLAST (i.e., BLASTp) analysis against *D. vulgaris* genome (a threshold of 10^{-20} was chosen for forward and backward expectation values). Notably, we also relaxed the irreversibility constraints on three existing reactions in the Stolyar's original model. All these reactions were reversible in other metabolic models. In addition, we added additional transport and exchange reactions to the model whenever required.

To work with a compact, yet efficient, representation of the *D. vulgaris* metabolism, we next included the new identified reactions from the KEGG in the model and solved a mixed-integer optimization problem to identify the minimum number of reactions from the draft Seed model that need to present in the corrected model to support growth in presence of lactate, acetate and ethanol. This analysis identified 32 reactions from the draft Seed model that should be kept in the model, where the rest can be removed. The final updated model contains 145 reactions and 119 metabolites. A brief comparison of the original Stolyar's model and the updated model for *D. vulgaris* is given in Table TS1.2. The complete list of reactions as well as the details of modifications to the model is given in Table S1 (Excel file).

Table TS1.2- Comparison of the Stolyar's metabolic model for *D. vulgaris* [9] and the updated model presented in this study.

	Stolyar's model [9]	***Updated model***
# of reactions	86	145
# of metabolites	82	119
Growth capabilities	Cannot support growth in presence of acetate or ethanol as carbon source	Supports growth in presence of acetate or ethanol as carbon source

References

1. Kumar VS, Maranas CD (2009) GrowMatch: an automated method for reconciling in silico/in vivo growth predictions. PLoS Comput Biol 5: e1000308.
2. Suthers PF, Zomorrodi A, Maranas CD (2009) Genome-scale gene/reaction essentiality and synthetic lethality analysis. Mol Syst Biol 5: 301.
3. Sahinidis NV (1996) BARON: A general purpose global optimization software package. Journal of Global Optimization 8: 201-205.
4. Segre D, Vitkup D, Church GM (2002) Analysis of optimality in natural and perturbed metabolic networks. Proc Natl Acad Sci U S A 99: 15112-15117.
5. Wintermute EH, Silver PA (2010) Emergent cooperation in microbial metabolism. Mol Syst Biol 6: 407.
6. Christensen BB, Haagensen JA, Heydorn A, Molin S (2002) Metabolic commensalism and competition in a two-species microbial consortium. Appl Environ Microbiol 68: 2495-2502.
7. Shrestha UT (2009) Microbial Association-Microbial Interaction.
8. Zhuang K, Izallalen M, Mouser P, Richter H, Risso C, et al. (2010) Genome-scale dynamic modeling of the competition between Rhodoferax and Geobacter in anoxic subsurface environments. ISME J.
9. Stolyar S, Van Dien S, Hillesland KL, Pinel N, Lie TJ, et al. (2007) Metabolic modeling of a mutualistic microbial community. Mol Syst Biol 3: 92.
10. Henry CS, DeJongh M, Best AA, Frybarger PM, Linsay B, et al. (2010) High-throughput generation, optimization and analysis of genome-scale metabolic models. Nat Biotechnol 28: 977-982.
